# Rapid Wide-Field Correlative Mapping of Electronic and Vibrational Ultrafast Dynamics in Solids

**DOI:** 10.1021/acsnano.4c15397

**Published:** 2025-02-10

**Authors:** Rihan Wu, Yaqing Zhang, Md Shahjahan, Elad Harel

**Affiliations:** Department of Chemistry, Michigan State University, East Lansing, Michigan 48823, United States

**Keywords:** pump−probe, ultrafast dynamics, wide-field imaging, hyperspectral imaging, electron−phonon coupling, heterogeneity, correlative mapping

## Abstract

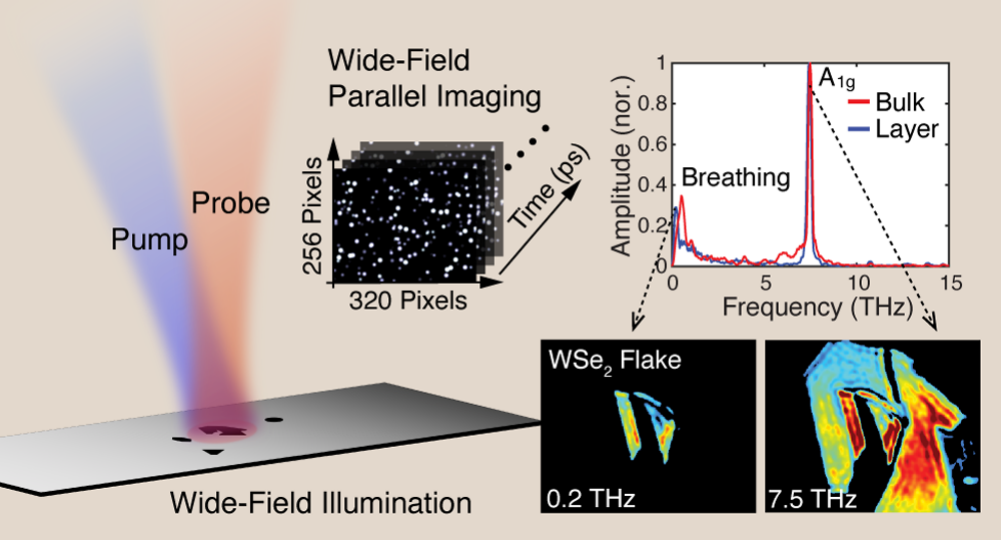

Coupling between electronic and vibrational degrees of freedom in solids is responsible for many fundamental material properties, including superconductivity, ferroelectricity, high thermal conductivity, and indirect bandgap emission among many others. In heterogeneous materials electronic-vibrational coupling gives rise to spatial correlations between the electronic relaxation properties and vibrational dynamics. Visualizing and mapping these correlations may lead to important physical insights for applications that include electronics, optoelectronics, and energy technologies. However, due to the vastly different energy scales involved, measuring and correlating electronic and vibrational properties is challenging. While in principle, ultrafast pulses with sufficient bandwidth generate excited-state population and vibrational coherence signatures, the need to measure the signal point-by-point across the sample results in relatively slow acquisition, leading to an increased risk of sample photodamage and rendering the measurements highly susceptible to noise. Here, we introduce Parallel Rapid Imaging with Spectroscopic Mapping (PRISM), an ultrafast, wide-field, and coherent imaging technique, that allowed for the simultaneous acquisition of electronic state decay in the 0–10 ps range and vibrational spectra in the structurally sensitive low-frequency 5–600 cm^–1^ range. The exceptionally high speed of PRISM, exceeding 1.6 million time-resolved traces per second, enabled the mapping of electronic and vibrational properties across 80,000 pixels simultaneously in few-layer tungsten diselenide and perovskite materials. Correlations between the population and coherence maps reveal spatial heterogeneity not observed by either measurement alone. The ability to map electronic-vibrational coupling makes PRISM particularly well-suited for fundamental studies of complex solids and a wide range of materials applications.

Electron–phonon coupling in solids is foundational to many critical phenomena in solid-state physics and materials science. For instance, superconductivity relies on electron–phonon interactions to facilitate the formation of Cooper pairs, enabling current flow without resistance.^1^ It also leads to the renormalization of electronic states, increasing electron effective mass, and the formation of polarons, in which electrons locally distort the lattice structure.^2^ Electron–phonon interactions manifest as changes in electronic state relaxation rates and the frequencies and dephasing times of lattice vibrations.^3^ Understanding the mechanisms that underlie these dynamics is essential for fundamental insights and the development of materials with tailored properties across applications in high-temperature superconductors,^4^ high-efficiency energy storage systems,^5^ advanced emission technologies,^6^ topological devices,^7^ quantum sensors,^8^ catalysis,^9^ and cell signaling networks.^10^

In materials with strong electron–phonon coupling, local property variations often arise from defects, strain, phase separation, or domain boundaries.^11^ Imaging and spectroscopic techniques that map these variations and correlate them with structural features are vital for understanding how microscopic interactions influence macroscopic behavior. This approach is especially valuable for probing emergent phenomena, where the interplay between electronic states and vibrational dynamics reveals the strength and nature of coupling locally. By measuring spatial correlations between carrier relaxation and phonon frequencies, it is possible to indirectly probe electron–phonon coupling in heterogeneous materials. However, many spectroscopic imaging techniques focus on either vibrational states,^12^ such as stimulated Raman scattering spectroscopy (SRS),^13,14^ coherent anti-Stokes Raman scattering spectroscopy (CARS)^15^ and two-dimensional infrared spectroscopy (2DIR),^16,17^ or population decay dynamics,^18^ as in two-dimensional electronic spectroscopy (2DES),^19,20^ time-resolved fluorescence microscopy (TRF),^21^ transient-absorption spectroscopy (TAS),^22,23^ pump–probe holographic microscopy^24^ and structured pump–probe microscopy (SPPM).^25,26^

Techniques capable of measuring both electronic and vibrational properties often face significant challenges due to the small changes in optical density (OD) associated with vibrational oscillations (10^–3^–10^–5^) compared to the electronic response (10^–2^–10^–3^). A commonly used approach for measuring both processes with high sensitivity is point-by-point scanning confocal pump–probe microscopy, where photodiodes combined with lock-in amplifiers (LIA) are typically employed for signal detection and isolation.^27−32^ LIA effectively isolates the signal by employing a narrowband filter to reject out-of-band noise, enabling the detection of subtle changes in OD induced by the pump laser. However, the necessity of scanning both in time and space significantly increases the acquisition time; capturing a full image stack at high spatial and temporal resolution typically requires several seconds to minutes.^27,32,33^ Although recent advancements employing acousto-optic (AO) devices have been developed to improve scan speed, a fundamental limitation remains due to the sound velocity within the AO crystal, restricting the acquisition time to a minimum of a few tens of microseconds per pixel.^27^ Prolonged exposure not only makes the signal more vulnerable to low-frequency noise but also increases the risk of photodamage, limiting the ability to observe short-lived phenomena such as phase transitions and nonrepeatable processes.^34^

Additionally, extended acquisition time may lead to a loss of spatial correlation due to sample drift or fluctuations, compromising the accuracy of correlation mapping. These limitations hinder the ability to fully capture the complexity of spatially dependent material properties, particularly in systems that undergo dynamical changes at short length scales where high spatial and temporal resolution is needed.

In this contribution, we introduce Parallel Rapid Imaging with Spectroscopic Mapping (PRISM), a nonscanning wide-field, ultrafast coherent imaging technique that enables simultaneous mapping of excited-state dynamics and vibrational motions with femtosecond precision and high spatial resolution. Data collection of an 81,920 pixels (250 × 320 pixels) image stack with >500 delay points can be completed in as little as 50 ms, resulting in over 1.6 million dynamic traces and wideband spectra (5–600 cm^–1^) per second. The effective acquisition time per pixel is approximately 600 ns, exceeding 2 orders of magnitude in efficiency compared to the fastest known scanning coherent pump–probe microscope with comparable spectral resolution,^27^ to the best of our knowledge, with potential for further enhancements using available camera and delay scanning technology, and advanced image analysis methods.

With respect to photon collection efficiency, PRISM is comparable to point-by-point scanning methods. However, for the latter, the need for both spatial and temporal scanning greatly limits the ability to suppress noise at each pixel and at each delay time. In contrast, the rapid acquisition speed of PRISM effectively suppresses low-frequency noise that accumulates along the time-scanning direction. We note that PRISM also fundamentally differs from previously reported wide-field pump–probe microscopy techniques in both speed and versatility. Wide-field methods developed so far mostly rely on lock-in cameras or EMCCDs.^35,36^ The slow frame rates, ranging from a few Hz to a few hundred Hz, render them incompatible with rapid delay line scanning. Therefore, these methods commonly adopt relatively slow point-by-point scanning of delay times, which, together with slow frame rates, extends the acquisition time to minutes or even hours. Similar to the point-scanning approach, the extended acquisition time increases the susceptibility of the signal-to-noise, limiting the detection of weak coherent vibrational signals. As a result, most reported wide-field pump–probe microscopy studies primarily focus on the electronic response.

In contrast, PRISM provides high-resolution mapping of both electronic and vibrational states, revealing the interplay between these states and their variations in relation to structural features. Detailed correlation analysis allows disentangling of local effects arising from sample morphology and uncovers hidden spatial domains where structural features influence electronic and vibrational dynamics. This capability makes PRISM well-suited for diverse applications, including material screening based on mechanical and chemical composition, defect analysis, real-time bioimaging, reaction pathway monitoring, and, more generally, tracking of irreversible events that occur on the millisecond to second time scale. Additionally, the extensive data sets (over a few GB per measurement) generated by PRISM may be used in advanced statistical analysis, including machine learning methods that may aid in uncovering hidden correlations and patterns in complex material properties and structures, which may offer new insights and optimizations for material design and discovery.

## Results

**Figure 1:**
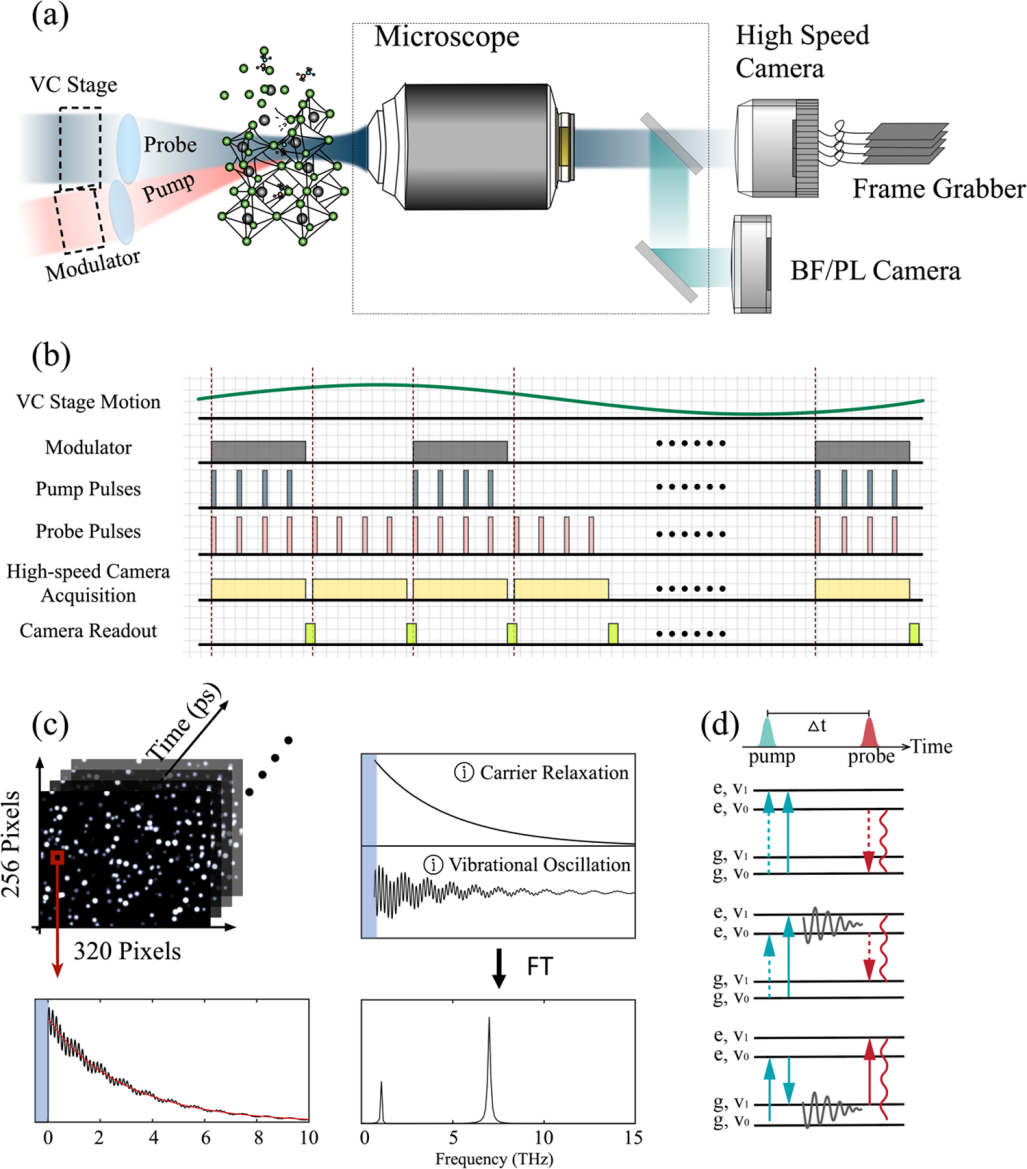
(a) Schematic of the PRISM setup. The pump and probe beams are softly focused onto the sample, with their arrival times controlled by a voice coil (VC) stage carrying a retroreflector in the probe path. An intensity modulator is placed in the pump path to block alternating beams, reducing background noise. (b) Timing diagram of the synchronized hardware components, including laser, camera, voice coil stage, and intensity modulator. (c) Conceptual sketch of a typical PRISM image sequence, where each pixel encodes local electronic and vibrational states information. (d) Energy level diagrams depicting three examples of pump-induced dynamics: excited state population, excited state coherence, and ground state coherence, respectively, followed by probe detection after a delay time Δ*t*.

The core concept of PRISM relies on the synchronized acquisition of time-resolved transmittance image sequences using a high-speed camera, capturing pump-induced changes frame by frame in real time. The pump and probe beams are softly focused to illuminate a wide field of view (FOV) of about 80 × 80 μm^2^ (Figure 1a). The transmitted probe beam was collected by a high-NA objective, and the scattered light from the sample was relayed to a high-speed camera capable of capturing up to 50 k frames per second (FPS). The time delay between the pump and probe pulses at the sample was controlled by a high-speed voice coil (VC) stage oscillating sinusoidally at 10 Hz along the probe optical path, varying the pump–probe arrival time while the camera simultaneously captured frames. To retrieve the differential transmission signal, a fast laser intensity modulator in the pump path operated at half the camera’s frame rate, blocking alternate pump pulses. The resulting image sequence, collected while the VC stage was in motion, was stored in a three-dimensional data cube *I* (*x*, *y*, *t*), where *x* and *y* represent spatial dimensions and *t* represents the pump–probe time delay. Additionally, bright-field (BF) and photoluminescence (PL) imaging were integrated into the system for correlative analysis, with the BF/PL camera capturing a single image at the beginning of each measurement. All hardware components were synchronized to ensure precise timing of signal generation, detection, and acquisition, which is critical for data averaging and enhancing the overall signal-to-noise ratio (SNR) (Figure 1b). Further experimental details are provided in the Methods and Supporting Information.

Figure 1c shows a conceptual sketch of a typical image sequence obtained from PRISM measurements. Each single pixel in the image sequence captures both the electronic state decay dynamics over pump–probe time delay, and high frequency coherent oscillations which may represent ground- or excited-state vibrations, or mixed electronic-vibrational (vibronic) quantum superpositions (Figure 1d). Analyses of the time-resolved image sequence allow for the retrieval of the lifetime of the electronic states, as well as the energy and dephasing rate associated with each vibrational coherence. To increase the computational efficiency, the 3D data cube was processed with a global analysis approach^37^ (detailed data analysis approach available in SI), which separated the high-frequency oscillatory signal from the low-frequency exponential decay. The oscillatory signal was subsequently transformed into the vibrational spectrum using a fast Fourier Transform (FFT), revealing distinct frequency components associated with different vibrational coherences.

To demonstrate the capability of the PRISM system, we performed measurements on three different materials, tungsten diselenide (WSe_2_), methylammonium lead bromide (MAPbBr_3_) and mixed iodide–bromide methylammonium lead (MAPb(Br_*x*_I_1–*x*_)_3_) perovskite crystal.

**Figure 2:**
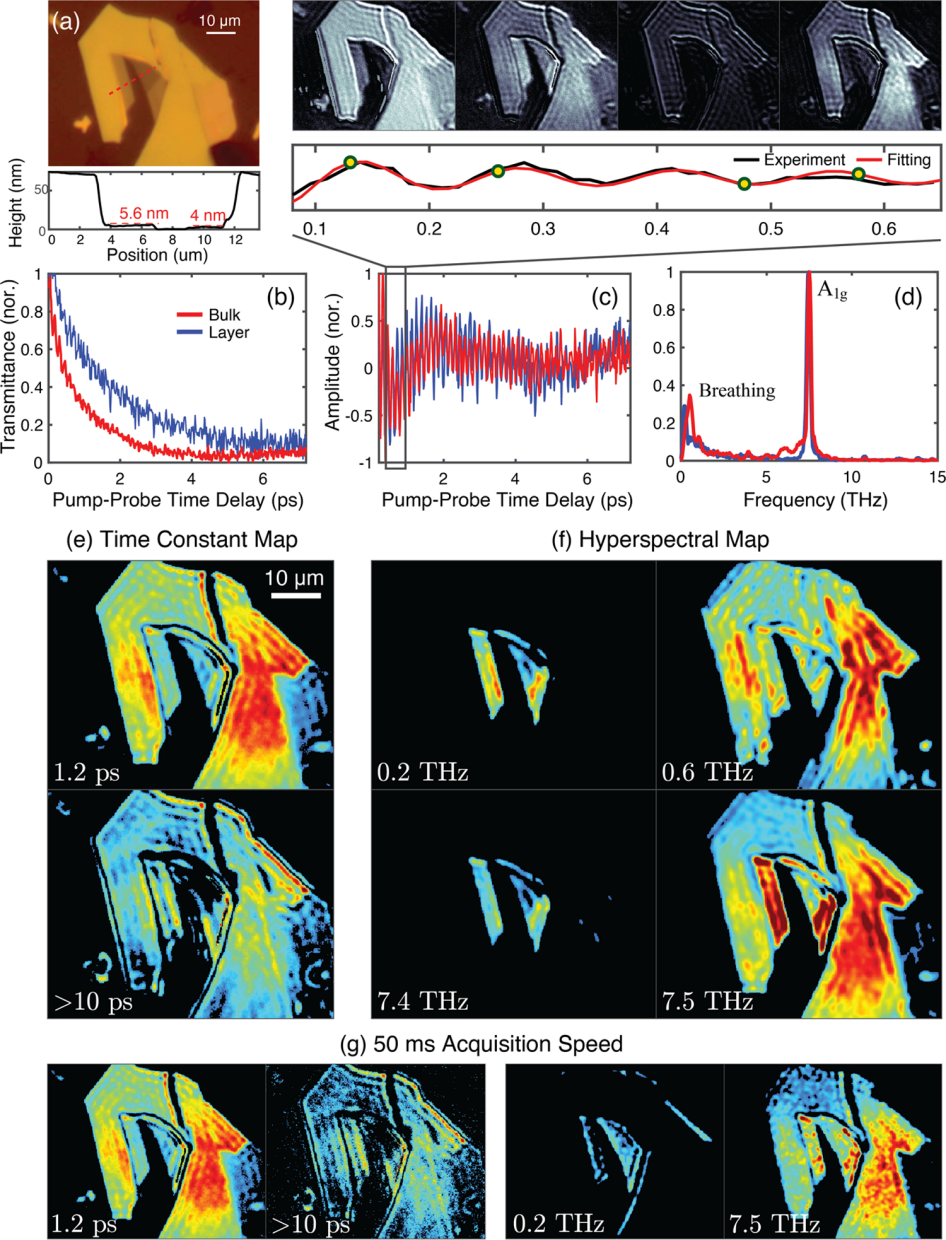
(a) Top: Bright-field image of a mechanically exfoliated WSe_2_ flake, showing a mixed morphology of bulk and few-layer regions; Bottom: AFM trace along the red dashed line. (b) Normalized transmittance of a single pixel from PRISM measurements in a layer (blue) and bulk region (red). (c) Isolated oscillatory components from (b). The zoomed-in 700 fs region highlights the sinusoidal nature of the oscillations. The image, after subtracting the exponential decay at the yellow point, reveals intensity variations across the entire flake. (d) Spectral comparison between the layer (blue) and bulk region (red). (e) Intensity map at two different decay time constants. (f) Hyperspectral map at four different frequencies, highlighting heterogeneities caused by variations in layer number. (g) Time constant and hyperspectral maps at highest acquisition speed of 50 ms.

Figure 2a shows the bright-field image of the WSe_2_ sample used in this study, where regions of few-layer thickness are surrounded by bulk material. With its distinct vibrational spectra, characterized by high-frequency intralayer vibrations and low-frequency interlayer vibrations,^38−40^ WSe_2_ served to demonstrate PRISM’s capability in identifying the influence of structural heterogeneity on molecular interactions. Atomic force microscopy (AFM) confirmed that the WSe_2_ bulk region’s thickness is about 72 nm, equating to around 100 layers given a monolayer thickness of ∼0.7 nm,^40,41^ whereas the few-layer regions measured 5.6 and 4 nm in thickness, corresponding to about 8 and 6 layers, respectively.

By tuning the pump wavelength to 750 nm with a spectral width of about 30 nm, we target the A-exciton absorption band of WSe_2_ near 760 nm,^39,42^ optimizing pump absorption efficiency and enhancing both carrier population and coherent phonon signal strength.^43^ When the pump energy exceeds the bandgap, coherent phonon excitation primarily occurs through the displacive excitation of coherent phonons (DECP) mechanism, where the spatial redistribution of optically excited electrons shifts the lattice equilibrium and generates coherent phonons.^44^ In this case, electrons and phonons are highly coupled, with the electronic redistribution directly influencing lattice vibrations (i.e., phonon coherences). Simultaneously, impulsive stimulated Raman scattering (ISRS) contributes to the phonon spectrum, although less prominently due to the inefficient two-photon process.^45,46^ The probe was tuned to an optimized wavelength of 650 nm with a spectral width of about 20 nm, balancing efficient absorption with sufficient separation from the pump wavelength.

Figure 2b–d compares the normalized transmittance as a function of pump–probe delay, the isolated oscillatory components, and the vibrational spectrum of a single pixel in the layer (blue) and the bulk region (red). Changes in both the decay dynamics and the vibrational spectrum can be observed as the sample thickness varies. In the oscillatory components shown in (c), both high-frequency and relatively low-frequency components are evident. Examining the first 700 fs, as highlighted in the black box above panel (c), reveals that the signal fits a decaying sinusoidal function, characteristic of typical vibrational oscillations. The images captured by the high-speed camera at different pump–probe time delays (yellow points), after subtracting the exponential components, reveal intensity variations across the entire flake caused by high-frequency vibrational oscillations.

To intuitively highlight this heterogeneity across the flake, we provide two sets of maps: the time constant maps (Figure 2e) and the hyperspectral maps (Figure 2f). Two decay components were identified during the global exponential fitting of the original data cube, with one time constant around 1.2 ps and another exceeding 10 ps. The intensity maps of these two components, shown in Figure 2e top and bottom, reveal the biexponential decay characteristics of WSe_2_ across the few-layer and bulk regions. We note that the decay time constant exceeding 10 ps, associated with the A-exciton lifetime,^47,48^ is identified throughout the fit, but the 7.2 ps delay scan range limits capturing the full decay curve, resulting in a larger uncertainty.

Figure 2f displays hyperspectral maps at four different frequencies. Distinct spatial features of few-layer and bulk are evident. In the sub-THz region, characterized by interlayer phonon modes, few-layer and bulk regions exhibit different intensity maps at frequencies of 0.2 THz and 0.6 THz, respectively. The 0.2 THz mode (∼6 cm^–1^) may be associated with the first order out-of-plane interlayer breathing mode in structures consisting of 6–8 atomic layers.^38^ Around 0.6 THz (∼20 cm^–1^), the bulk region exhibits a stronger signal, which can be attributed to interlayer shear motion within the bulk material.^49^ We note that the frequency resolution limit of 0.14 THz introduces uncertainty in determining the exact center frequencies. In the high-frequency region, the peak near 7.5 THz is attributed to the A_1g_ phonon mode.^39,50^ Other Raman active modes such as E^1^_2g_, B^1^_2g_ and LA(M),^50,51^ have insignificant contribution to the spectrum, likely because the DECP mechanism is dominant, resulting in fully symmetric modes having significantly stronger intensity.^45,52^ The coherence maps reveal that both the few-layer and bulk regions exhibit frequency components at 7.5 THz, whereas only the few-layer region displays the 7.4 THz components. This redshift and broadening of the A_1g_ mode can be attributed to increased electron–phonon coupling, resulting from weakened interlayer interactions and lattice softening induced by quantum confinement effects.^39,53^ The reduction in interlayer coupling makes phonon modes more responsive to electronic excitations, while quantum confinement further intensifies this interaction by increasing the overlap between electronic states and phonon vibrations.

To validate the PRISM results on WSe_2_, we conducted a comparative study using a point-by-point scan spontaneous Raman instrument with a 785 nm continuous wave (CW) excitation source. Figure S6 in SI illustrates the comparison between the single-pixel spectrum and the overlaid hyperspectral maps obtained from both the PRISM and spontaneous Raman setups. The results show that, despite the lower spatial resolution and sensitivity of the point-by-point Raman measurements, the single-pixel spectrum aligns well with the PRISM data. This consistency confirms PRISM’s accuracy in capturing vibrational dynamics.

We note that the measurements in Figure 2b–f were signal-averaged 20 times to enhance the SNR, resulting in a total acquisition time of 1 s. However, as previously noted, a full PRISM measurement can be completed within 50 ms. The resulting time constant and hyperspectral maps are presented in Figure 2g (for a comparison of results obtained at different acquisition times, refer to the Figure S7 in SI). The time constant maps, which primarily reflect exponential decay dynamics, exhibit minimal variation with reduced acquisition times. In contrast, the hyperspectral maps show greater sensitivity to changes in acquisition time. Nevertheless, both the time constant and hyperspectral maps reveal the decay and vibrational characteristics consistent with the averaged results. Further improvements on acquisition speed are achievable through both hardware and software advancements, such as faster cameras, enhanced delay control mechanisms, and the application of advanced image denoising techniques.^54,55^

Next, we conducted PRISM measurements on solution-processed MAPbBr_3_ and MAPb(Br_*x*_I_1–*x*_)_3_ perovskite microcrystals. Solution-processed perovskite materials are known to exhibit inherent heterogeneity, including variations in composition, morphology and structural properties.^56^ It has been suggested that in metal halide perovskites, excellent carrier mobility and extended lifetimes result from large polaron formation, driven by electron–phonon coupling as electrons polarize and distort the soft lattice.^57^ This electron–phonon interaction is highly sensitive to the microenvironment, where local variations influence coupling strength and alter the spatial distribution of charge carriers, ultimately affecting key optical properties such as absorption and PL efficiency.^58^ While most studies focus on heterogeneity between different crystals, we selected these two examples to demonstrate PRISM’s ability to evaluate heterogeneity within a single crystal. Additionally, perovskites at room temperature typically present a broad vibrational spectrum^59,60^ at relatively low frequency which is difficult to measure using other spectroscopic imaging methods such as CARS or ISRS.

**Figure 3:**
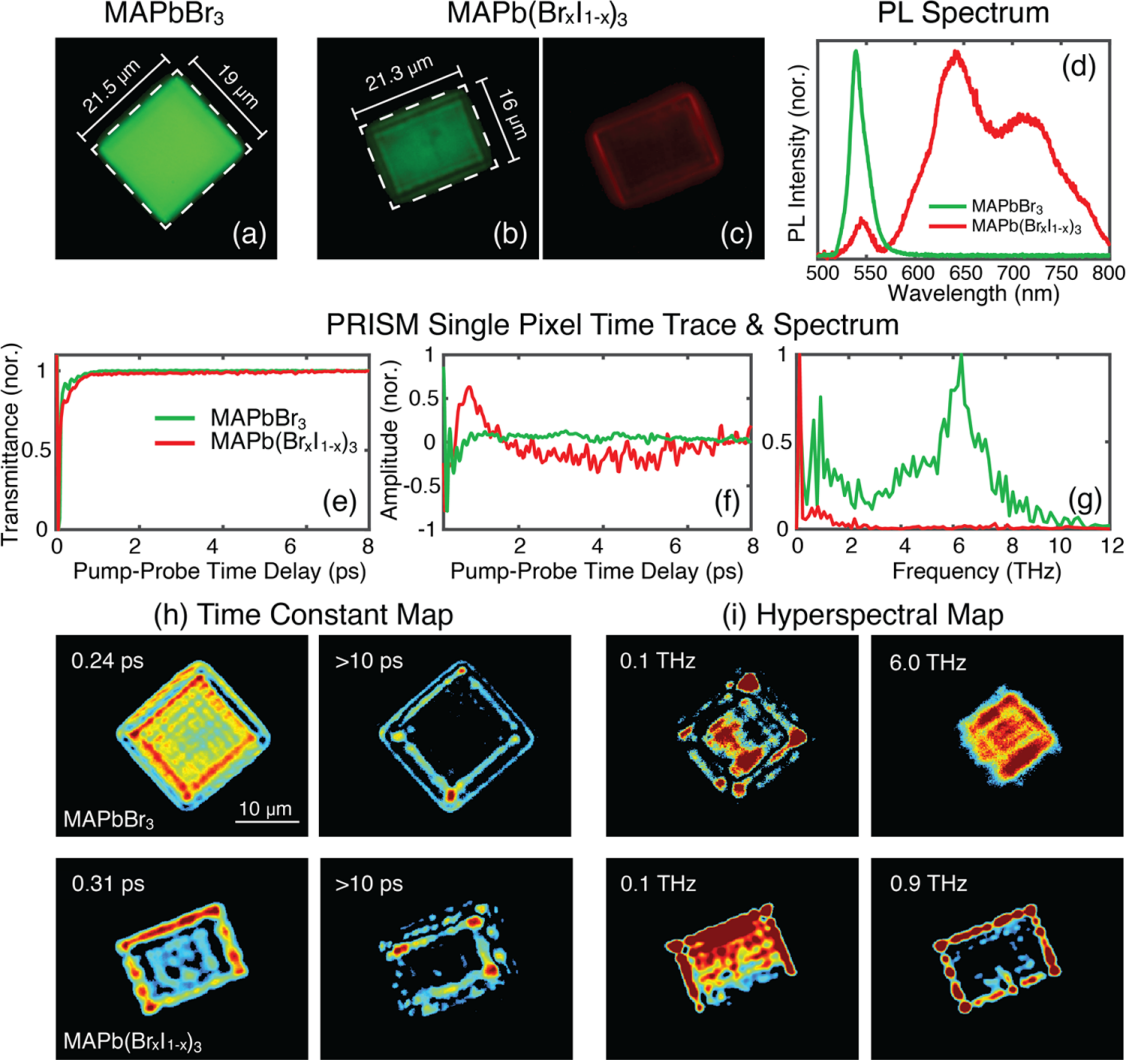
(a–c) PL images of solution-processed MAPbBr_3_ and MAPb(Br_*x*_I_1–*x*_)_3_ microcrystals, captured using a CMOS camera with fluorescence filters centered at 550 nm (green) and 650 nm (red). (d) Integrated PL spectra of both crystals. (e) Normalized transmittance changes as a function of pump–probe delay time at a single pixel in the PRISM images, taken at the crystal center. (f) Isolated oscillatory signal from (e). (g) Spectrum comparison between the two types of crystals. (h) Decay time constant maps for MAPbBr_3_ (top) and MAPb(Br_*x*_I_1–*x*_)_3_ (bottom). (h) Hyperspectral maps highlighting crystal heterogeneity.

Figure 3a–c shows PL images of MAPbBr_3_ and MAPb(Br_*x*_I_1–*x*_)_3_ crystals, captured at the start of each synchronized PRISM scan. The PL spectra of the two types of crystals are shown in Figure 3d. For MAPbBr_3_, the PL peak is centered around 540 nm, while partial substitution of bromide ions with iodide leads to a redshift in the bandgap, resulting in a broader PL wavelength distribution in the 600–800 nm range.^61−63^Figure 3e shows the normalized time-resolved transmittance change of a single pixel located at the center of the crystal from the PRISM measurements. It is evident that replacing part of the bromide atoms with iodide alters the time-resolved response. The isolated oscillatory components shown in Figure 3f, highlight distinct vibrational dynamics between the two types of perovskites, with these differences further emphasized in the spectra shown in Figure 3g. For MAPbBr_3_, two broad peaks are identified, spanning the 0–2 THz and 4–9 THz regions. The 0–2 THz peak corresponds primarily to lattice vibrations (TO/LO phonons), while the higher-frequency peak is associated with the torsional motion of the methylammonium (MA) cation, coupled to the halide cage.^60,64^ In MAPb(Br_*x*_I_1–*x*_)_3_, the 4–9 THz mode is significantly quenched, likely due to the altered bond strength and bond length within the halide cage. This quenching could also be attributed to the resonant pump excitation in MAPb(Br_*x*_I_1–*x*_)_3_, where the low-frequency modes are coupled with the electronic excitation, leading to modifications in the intensity of the high frequency peak.^65^

As indicated by Figure 3a–c, in single-crystals, heterogeneity is not easily discernible through PL imaging. In contrast, PRISM measurements, as shown in Figure 3h,i, reveal spatial variations in both the electronic and vibrational properties within the single crystal. The decay time constant intensity map in Figure 3h identifies two decay components: a fast component (<0.5 ps), attributed to hot carrier thermalization to the band edge,^66^ and a slower component (>10 ps) that appears primarily near the crystal edge, potentially indicating recombination through surface states caused by local strain-induced lattice distortions. We note that our measurements focus on the first few picoseconds after time zero to resolve phonon oscillations and dephasing; the slow recombination channels that typically occur on the nanosecond scale^67^ in perovskites are treated as a constant during the fitting process. The hyperspectral maps in Figure 3i further demonstrates the microcrystal’s heterogeneity. In MAPbBr_3_ crystals, a low-frequency phonon mode associated with lattice motions is detected at the crystal edge, while a higher-frequency mode around 6 THz, attributed to MA cation motions, is more pronounced at the crystal center. In contrast, for MAPb(Br_*x*_I_1–*x*_)_3_ crystals, the low-frequency mode near 0.1 THz appears uniformly throughout the crystal, whereas the 0.9 THz mode is localized specifically at the edges. The edge phonon mode in both types of crystal likely arise from lattice distortions at the edge termination. For MAPb(Br_*x*_I_1–*x*_)_3_ this mode may also reflect compositional variations due to phase segregation, which often initiates at grain boundaries.^68^ Moreover, the distinctive characteristics of electronic decay and the phonon mode at the edges, compared to the crystal center, suggest that different types of polarons may form at or near this region. Further investigation, which is beyond the scope of this study, is required to fully understand these phenomena. The comparison of the single-pixel time-resolved response at the crystal center and edge is provided in the SI.

## Discussion

PRISM’s ability to capture both carrier and vibrational dynamics simultaneously paves the way for exploring correlations between these properties. Such correlation can reveal changes in relaxation and recombination rates due to carrier-phonon coupling, as well as spectral shifts resulting from carrier-induced lattice distortions. To conduct this correlative analysis, it was crucial to distinguish subtle spatial variations in decay constants. Therefore, a point-by-point fitting of the exponential decay terms was performed to capture the variance in the parameters across different regions. The results show that in the few-layer region, the decay time constants exhibit a broader range (1.0–1.5 ps) compared to the bulk region (1.2–1.4 ps) (Figure S9 in SI). This broader distribution in the few-layer region may arise from surface and defect states introducing additional relaxation pathways, as well as the presence of multiple phonon modes with varying coupling strengths, leading to a more diverse range of decay times.^69^

**Figure 4:**
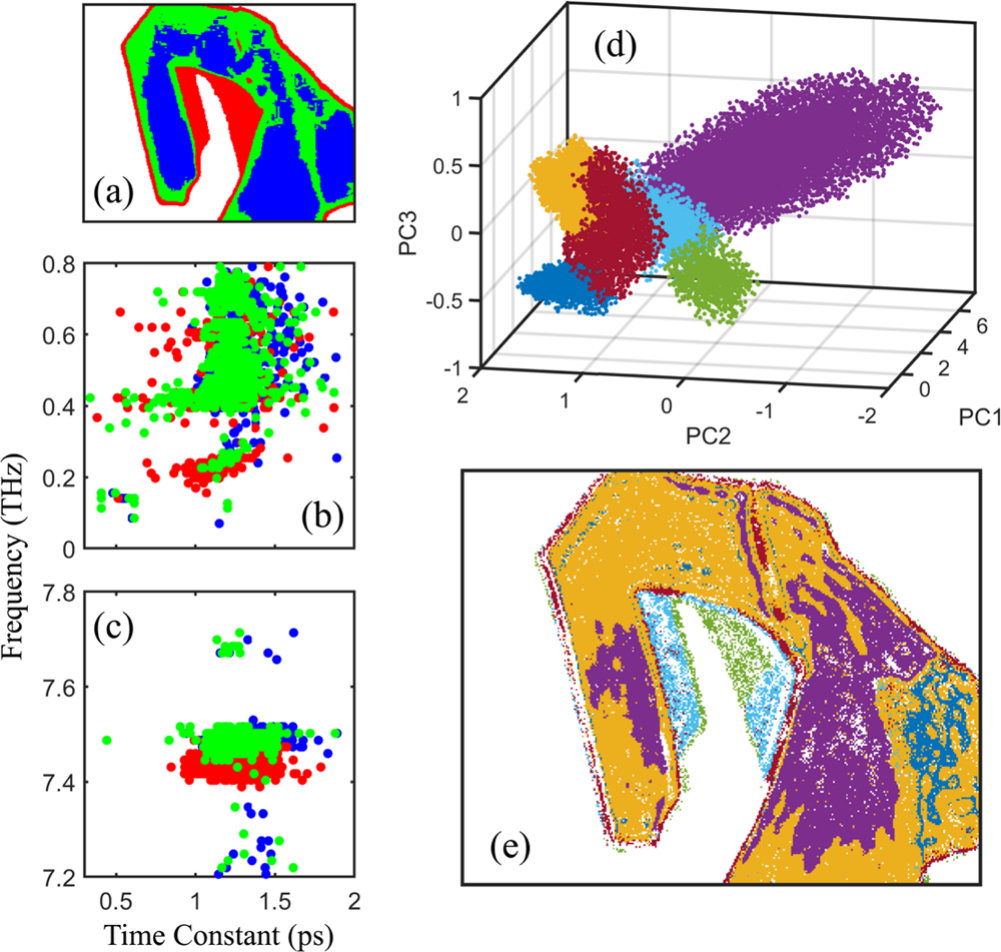
(a) Segmentation of different regions based on bright-field transmittance. The red region primarily corresponds to few-layer areas and edges, while the blue and green regions represent bulk areas. (b) Correlation scatter plot for the low-frequency vibrational region. (c) Correlation scatter plot for the high-frequency vibrational region. (d) Principal component analysis based on seven parameters: two decay components (∼1.2 ps and >10 ps), four spectral peaks (0.2, 0.6, 7.4, and 7.5 THz), and bright-field transmittance. (e) Spatial mapping of regions corresponding to the clusters identified in (d).

We further correlated the PRISM results with bright-field image (see SI for image registration). Bright-field microscope can distinguish WSe_2_ layers up to a few layers thick, as each additional layer significantly alters light absorption, refractive index, and interference effects, creating visible contrast.^70^ However, as thickness increases, probing morphological changes in these regions becomes increasingly challenging, necessitating the use of alternative techniques such as AFM. Here, we integrated bright-field microscopy with PRISM to provide a complementary approach for enhanced heterogeneity characterization. Based on the bright-field transmittance, we segmented the sample into three distinct regions, as shown in Figure 4a, and correlated the decay time constants with vibrational frequencies (Figure 4b,c), revealing signal clustering corresponding to specific spatial components. In these regions, the red area primarily represents the few-layer region and edges, while the blue and green regions correspond to bulk areas. Along the decay time axis, phonon peaks cluster around a 1.2 ps time constant, consistent with the global analysis results. The average decay time constants for the three regions are 1.23 ps (red), 1.3 ps (green), and 1.34 ps (blue), respectively. For vibrational modes, clustering is observed in the low-frequency domain, with peaks around 0.2 THz and 0.5–0.7 THz across the different regions. In the high-frequency range, the phonon mode in the few-layer region shows a slight red shift at 7.43 THz compared to 7.48 THz in the bulk region. These observations are consistent with the trends identified in Figure 2, further highlighting the spectral differences between few-layer and bulk dynamics.

While subtle differences between regions were observed, the decay rates and phonon frequencies showed strong overlap across spatial regions, making it challenging to disentangle dynamic effects influenced by sample heterogeneity. To further reduce the data dimensionality and reveal correlated variables, we conducted principal component analysis (PCA) based on seven variables: the fast and slow decay components, four specific spectral peaks (0.2, 0.6, 7.4, and 7.5 THz), and bright-field transmittance (see SI for details). As shown in Figure 4d, distinct clustering of data along the first three principal components can be observed. Based on this analysis, the sample was classified into six regions (Figure 4e), representing local heterogeneity likely arising from variations in sample morphology, such as differences in thickness, surface termination, localized strain, or the presence of defects. The green and blue regions correspond to few-layer areas of varying thickness, while the yellow, purple, and dark blue regions represent bulk areas. This segmentation not only highlights differences across distinct layers but also uncovers variations within layers that may appear homogeneous using other characterization techniques. Additionally, distinct types of edges are observed, one associated with the bulk region and another with the few-layer region, indicating sharp transitions in material properties at these boundaries.

Closer examination reveals that the purple cluster extends significantly along the PC1 and PC2 axes (see projection and PCA score vector in SI), despite its similarity to the yellow region in the bright-field image. However, its distinct distribution in PCA coordinates demonstrates notably different electronic and vibrational dynamics. The score vector for this cluster predominantly reflects contributions from slow decay components and phononic activity (Figure S10b in SI), with a stronger presence of these features suggesting enhanced phonon–electron coupling between the two states. The enhanced coupling could result from strain, localized defect states (e.g., vacancies, dislocations, grain boundaries), or surface chemistry introduced during the mechanical exfoliation process. Strain-induced effects may involve modifications to the bandgap energy, band alignment, lattice constants, and the softening or splitting of phonon modes.^71−74^ Depending on the strain type (e.g., uniaxial, tensile, or compressive), electron–phonon coupling may either increase or decrease. Similarly, localized defect states and surface chemistry may also contribute to the heterogeneity observed in the electronic and vibrational properties. By effectively distinguishing these regions within PCA space, PRISM uncovers diverse inter- and intramolecular interactions in visually homogeneous areas that are challenging to detect with conventional measurements. Techniques such as Raman spectroscopy, AFM, second-harmonic generation (SHG) imaging, and scanning transmission electron microscopy (STEM) have been employed to characterize these local effects but often face challenges such as indirect measurements, long acquisition times, restrictive environments, and invasive measurements.^75^ The ability to observe local variations in phonon–electron coupling, excitonic behavior, and overall electronic and optical properties is important for applications such as optoelectronics, photonic circuits, photovoltaics, flexible electronics, and light-emitting devices. PRISM provides an effective approach to capture and analyze these effects directly and more efficiently.

In summary, using WSe_2_ and perovskite microcrystals, we have demonstrated PRISM’s capability to resolve structural heterogeneity and correlate electronic and vibrational dynamics. By enabling simultaneous mapping of carrier and lattice behaviors across large areas, PRISM offers the potential for studying electron–phonon coupling in a variety of materials, extending beyond the examples in this work. Its wide-field, nonscanning approach, combined with fast acquisition speeds, addresses the limitations of traditional point-by-point methods, allowing for spatial-temporal-spectral correlation and integration with both imaging and nonimaging techniques. This flexibility makes PRISM suitable for monitoring both static and dynamic processes in various physical, chemical, and biological contexts.

## Methods

### PRISM Setup

In the wide-field imaging measurement, the pump and probe beams were generated by a second harmonic and a third harmonic noncolinear optical parametric amplifier (Light Conversion Ltd.), respectively. Both beams featured a repetition rate of 200 kHz and pulse duration of ∼32 fs (pulse characterization details can be found in SI). The pump beam had an adjustable wavelength range of 680–900 nm, while the probe beam’s range was tunable from 530 to 730 nm. The pump and probe beams were softly focused by a curved mirror to the sample plane with a FOV of 80 × 80 μm^2^. The pump beam was directed at the sample at an angle of ∼10°, while the probe beam was aligned with the optical axis. This noncollinear geometry caused a slight spatial variation in the effective time-zero across the 50 μm field of view due to differences in the optical path lengths of the pump and probe beams, resulting in a calculated time-zero difference of approximately 14 fs. Since this variation was small compared to the temporal resolution of the experiments, no explicit correction was necessary. The delay time between the pump and probe was controlled by a voice coil stage (Physik Instrumente) placed in the probe path, oscillating sinusoidally at 10 Hz with a maximum time delay of 7 ps. This range can be extended to hundreds of picoseconds by introducing multiple reflections on the retroreflector mounted on the stage, albeit with a trade-off in time resolution, making it suitable for studying low-frequency oscillations, carrier relaxation, and diffusion processes. An 50 × /0.42 NA objective (Mitutoyo) collected the transmitted probe light through the sample, forming the wide-field image through a tube lens and subsequently captured by a high-speed camera (Phantom S710) at a frame rate of 20,000 FPS and readout by a frame grabber (Euresys) while the stage was in motion. The camera featured a resolution of 256 × 320 pixels and with the FOV tailored to 57.5 × 46 μm. We note that, with a camera frame rate 10 times lower than the laser repetition rate, 10 laser pulses were binned per frame. The fast-scanning stage introduces a time difference of approximately 1 fs between successive laser pulses, resulting in a delay time smearing of about 10 fs, which is below the laser pulse duration. Therefore, this effect is negligible in comparison to the PRISM time resolution. A high-speed intensity modulator (Thorlabs OM6ENH) was used to block alternate camera frames, enabling a frame subtraction scheme to suppress 1/f noise as well as laser and environmental fluctuations. The real-time position of the stage was monitored using an interferometric method. The beam emitted from a Helium–Neon (HeNe) laser was split into two paths, with one passing through the voice coil stage and the other being reflected by a fixed mirror. The interference pattern, resulting from the combination of the two reflected beams, was detected by a photodiode (Thorlabs) and recorded by a high-speed digitizer (AlazarTech). The laser, camera and digitizer were synchronized to the same electronic pulse train generated by a counter output module (National Instruments). The full synchronization scheme can be found in SI. In addition, bright-field (BF) and photoluminescence (PL) images were acquired using an integrated CMOS camera (Thorlabs) within the PRISM system, synchronized with all the hardware. A single image was captured at the start of each scan.

### Sample Preparation

The WSe_2_ used in this study was prepared through mechanical exfoliation using Scotch tape. Thin layers of WSe_2_ were lifted from a bulk crystal and transferred onto a clean glass substrate by pressing the tape onto the surface and gently peeling it away, leaving behind exfoliated flakes. The MAPbBr_3_ and MAPb(Br_*x*_I_1–*x*_)_3_ mixed halide microcrystals were synthesized with a spontaneous solvent evaporation method. Precursor solutions for MAPbBr_3_ were prepared by dissolving equimolar amounts of MABr and PbBr_2_ in dimethylformamide at room temperature, followed by stirring the mixture at 1000 rpm for 2 h to ensure complete dissolution. Similarly, MAI and PbI_2_ were dissolved in Gamma-Butyrolactone (GBL) to form the MAPbI_3_ precursor solution. The mixed halide microcrystals of MAPb(Br_*x*_I_1–*x*_)_3_ were obtained by mixing the MAPbBr_3_ and MAPbI_3_ precursor solutions in a 10:3 ratio. For crystallization, the precursor solution was further mixed with GBL in a 1:1 ratio to produce a saturated solution. A microdroplet (∼1 μL) of the supernatant was then placed on a cover glass, and upon natural solvent evaporation, microcrystals began to form within minutes.

## Supporting Information Available

Experimental details of PRISM; hardware synchronization scheme; pump and probe pulse characterization before the microscope and at the sample plane; global fitting and data analysis; comparison between PRISM and point-by-point scanning Raman; results comparison at different acquisition speeds; single pixel time traces at perovskite microcrystal edge and center; correlation and principal component analysis; and the effect of sample thickness on signal level (PDF)
