# Supplementary material for: Rapid Wide-Field Correlative Mapping of Electronic and Vibrational Ultrafast Dynamics in Solids
Source: ACS Nano. 2025 Feb 10;19(7):7064–74. doi: 10.1021/acsnano.4c15397 (PMC11867015; doi:10.1021/acsnano.4c15397)
Supplement: Supplementary file 1 — nn4c15397_si_001.pdf [file nn4c15397_si_001.pdf]

Supplementary Materials for

Rapid Wide-field Correlative Mapping of Electronic and Vibrational Ultrafast Dynamics in Solids

Rihan Wu, Yaqing Zhang, Md Shahjahan and Elad Harel∗

Department of Chemistry, Michigan State University, East Lansing, MI 48823

E-mail: elharel@msu.edu

Experimental Details

In the PRISM setup a second harmonic noncollinear optical parametric amplifier (2H NOPA) is used as the pump pulse source and a third harmonic (3H) NOPA as the probe source. Both the 2H and 3H NOPAs are pumped by the same 1028 nm femtosecond laser source with a repetition rate of 200 kHz. 35 mW pump power was delivered to the sample location with a spot diameter of approximately 80 μm, resulting in a fluence of approximately 7 mJ/cm^2, which is comparable to that used in other studies[1,2]. For the probe, we employed a power of 3 mW, maintaining the same spot size, resulting in a fluence of 0.6 mJ/cm^2. Both the transmitted pump and probe beams are collected using a 50X objective lens with a numerical aperture of 0.42 (Mitutoyo Plan Apochromat). Subsequently, the pulses are separated by a 700 nm short pass dichroic mirror. The pump beam is directed toward a photodiode for monitoring purposes, while the probe beam is sent through a 200 mm tube lens. The resultant image at the intermediate focal plane is then relayed to a high-speed camera (Phantom S710) using a pair of lenses with focal lengths of 50 mm and 100 mm, respectively. The total magnification factor of the setup is approximately 100X, designed to match the 20 μm pixel size of the camera. The theoretical field of view (FOV) in the sample plane, with a 256×320 pixels camera sensor, is

51×64 μm. We characterized the actual FOV using a microscope calibration slide, resulting in measured dimensions of 46×57.5 μm. The probe power is adjusted to ensure that the camera operates near saturation with an exposure time of 50 μs (corresponding to a frame rate of 20 kHz) in the absence of a sample. This ensures full utilization of the camera's dynamic range, maximizing signal intensity relative to camera noise.

A retroreflector mounted atop a high-speed voice coil stage operating at 10 Hz was positioned in the probe optical path to dynamically modulate the optical path length while the camera captures frames at rate of 20 kHz. Consequently, the pump-induced change in the probe transmission through the sample at the full range of pump-probe time delays requires 1000 frames within the camera image sequence. As the stage moves sinusoidally, one full scan of the pump-probe delay corresponds to a half-period of stage motion, resulting in an acquisition time of 50 ms. Since the stage movement is not linear, while the camera acquisition occurs at regular intervals, the time-domain signal is non-linearly sampled in the molecular time frame (i.e., the pump-probe delay time). As a result, linear resampling is performed on the data prior to analyzing the decay time constants or vibrational oscillation frequencies. The linear correction requires the stage position to be known at each camera frame. Due to the rapid movement of the delay stage, its position feedback accuracy falls short of the experimental requirements. Therefore, the real-time position of the stage is monitored using an interferometric method with a Helium-Neon (HeNe) laser. The laser beam is split into two paths: one passes through the voice coil stage while the other is reflected by a fixed mirror. The interference pattern, resulting from the combination of the two reflected beams, is detected by a photodiode and recorded by a high-speed digitizer (AlazarTech) at a sampling rate of 1 M/s. As the stage moves, it changes the path length of one arm of the interferometer, leading to a shift in the interference pattern, which in turn affects the intensity detected by the photodiode. At every half period of HeNe

wavelength, the stage moves a distance that represents 0.53 fs in molecular time. The interferometric method, therefore, has sufficient time resolution to accurately assign the correct pump-probe delay for each recorded frame. The relationship between the lab time frame and the molecular time frame is captured by a calibration curve at each stage cycle as shown in Figure S1. Details of the resampling process based on this calibration procedure are described in the next section. While the nonlinear movement of the stage adds some complexity to the data analysis, it also offers certain advantages. Around the stage's turning point, where the velocity is nearly constant, the dynamic trace is densely sampled by the camera. By adjusting the time zero to be near this turning point, the camera finely samples the pump-induced changes occurring at early times, which may include rapid electronic responses and strong vibrational oscillations. This approach effectively broadens the detection bandwidth and enhances the signal-to-noise (SNR) ratio for high-frequency components. To further improve the SNR, we implemented a frame subtraction scheme, utilizing a high-speed modulator (Thorlabs OM6ENH) to effectively chop the pump intensity at half of the camera's frame rate (10 kHz). The modulator and camera are synchronized, ensuring the camera captures alternating frames with and without pump excitation. Subtracting these alternating frames then yields the differential transmission signal at each pixel, thereby isolating the pump-induced changes and enhancing the SNR by mitigating the influence of background noise.

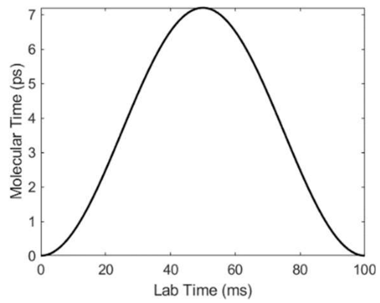

Figure S1. Calibration curve between the lab time (camera frame time) and molecular time (stage position).

We have implemented a comprehensive timing scheme to synchronize all the hardware components, ensuring they operate on a common clock. This integration includes the high-speed digitizer for monitoring the HeNe signal, pump modulator for chopping, camera, laser source and voice coil stage. A compact data acquisition system (c-DAQ, National Instruments),

featuring three frequency generation channels and two analog output channels, is employed to generate the clock, trigger TTL signals, and provide analog control for the stage and modulator. All these channels operate based on the same on-board clock, ensuring precise timing. The frequency generation channels produce 3.3 V TTL signals at 1 MHz, 200 kHz, and 20 kHz, which are used for the digitizer clock, laser control, and acquisition trigger, respectively. One of the analog channels generates a sinusoidal signal with a 10 V amplitude to control the stage, while the other generates a 1 V square wave signal for modulator control. As the experiment begins, the analog control signals are sent to the stage and modulator a few seconds before the acquisition to ensure the mechanical stability of these components. Subsequently, an acquisition trigger is sent to the camera and digitizer to initiate data recording. The camera captures each frame upon receiving a pulse from the 20 kHz TTL pulse train. A basic outline of the synchronization pulse sequence is shown in Figure S2.

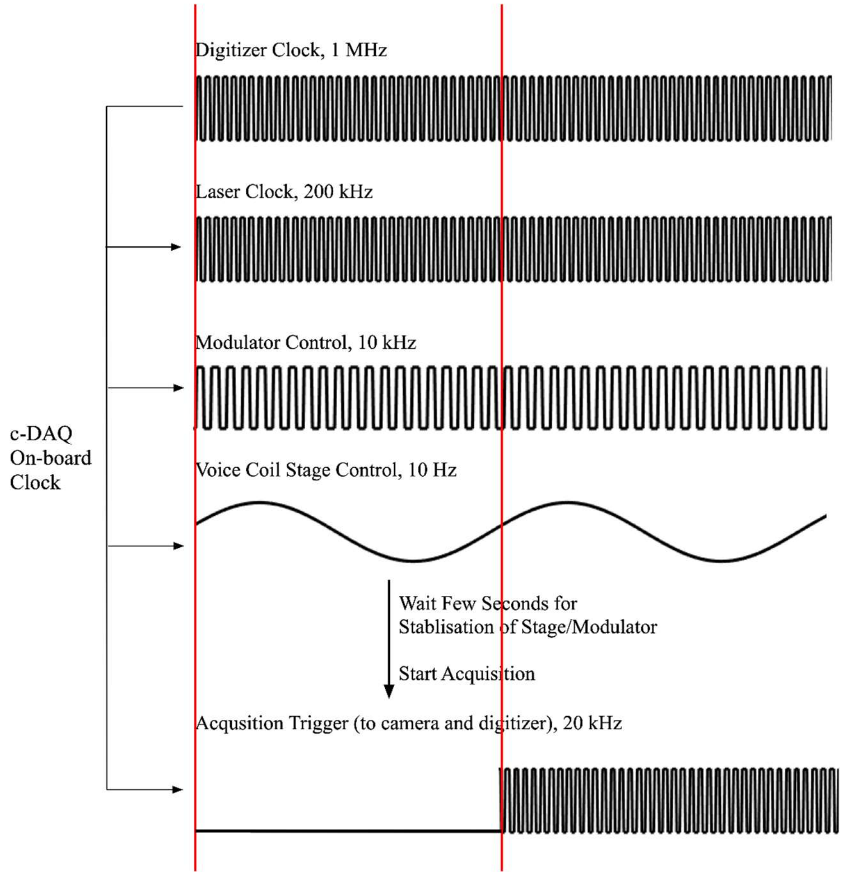

Figure S2: c-DAQ output pulses for synchronization of the PRISM hardware.

Pulse Characterization

To achieve high temporal resolution and sufficient spectral bandwidth, ultrashort pump and probe pulses were used in this work. The pulse durations were carefully characterized both before entering the microscope and at the sample plane. Prior to entering the microscope, the pulses were measured using an autocorrelator. The autocorrelation trace exhibits a full width at half maximum of about 46 fs (Figure S3(a)), corresponding to a pulse duration of approximately 32 fs for both pump and probe beams. Inside the microscope, we used four low-GDD mirrors to deliver the pump and probe beams and focused both beams using curved mirrors to avoid dispersion induced by transmissive optical components. To verify the pulse duration at the sample plane, we collected the pump-probe response of a silica substrate in the absence of a sample (XPM measurement). As shown in Figure S3(b), the measured trace closely matches the simulated XPM trace[3] with pulse duration of ~40 fs, confirming that both pulses were not stretched much inside the microscope.

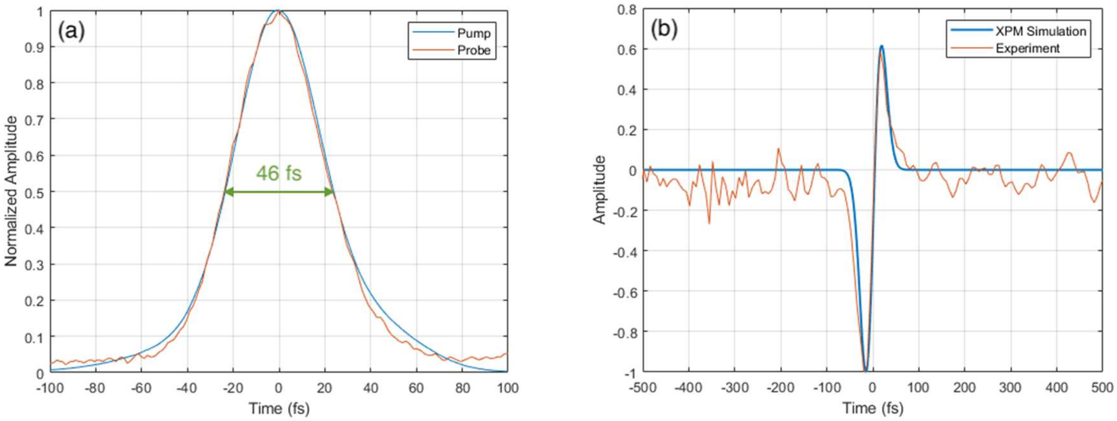

Figure S3: (a) Autocorrelation trace of the pump and probe pulses measured before entering the microscope. The full width at half maximum (FWHM) is approximately 46 fs, corresponding to a pulse duration of about 32 fs. (b) Measured pump-probe response of silica substrate at the sample plane (red) and the simulated XPM trace with pulse duration of 40 fs

(blue).

Data Analysis

The first step of the data analysis is image reconstruction. Due to the high frame rate of the camera, the data is continuously streamed in parallel through 16 CoaXPress channels at a speed of up to 7Gpx/s. This parallel streaming approach differs from conventional CMOS cameras, which typically stream data sequentially, allowing for straightforward image reconstruction. To facilitate this parallel streaming, the sensor area is divided into four banks labeled A, B, C, and D. Each bank, comprising 256×80 pixels, streams data independently. Two eight-channel frame grabbers (Euresys Coaxlink-Octo) simultaneously retrieve the data streams from these banks, temporarily storing them on the PC's random-access memory, and then writing them to disk. Image reconstruction is subsequently carried out in Matlab. For the upper half of the image, the reconstruction process follows the bank order of D, C, B, A, line by line (256×1 pixels), while for the lower half of the image, the order is reversed. This reconstruction is performed for all the frames in the experimentally obtained image sequence.

Next, subsequent frames are subtracted to isolate the pump-induced signal only. It is important to note that this process results in a reduction of the experiment's time resolution by half. Following the steps of image reconstruction and frame subtraction, the image sequence is organized into a format of 256 pixels in width, 320 pixels in height, and 500 frames along the time dimension. For the results presented in the main text, we performed 20 continuous scans of the pump-probe delay (10 stage period, 1 s acquisition time), resulting in 20 independent image sequences. For all the data shown in the main text except for Figure 2(g), we averaged these image sequences together after completing the reconstruction and subtraction processes

to enhance the signal-to-noise ratio and improve data quality.

The next step in data analysis is the linear resampling along the time dimension of the data hypercube. As mentioned, the data is initially not uniformly distributed in molecular time. To correct this nonuniform sampling, we first conducted a Fast Fourier Transform (FFT) along the time dimension of the data, followed by zero-padding in the frequency domain. This increased the time point density after an inverse FFT back to the time domain. Utilizing the previously measured calibration curve, we estimate the pixel amplitude at each linearly spaced point in molecular time through linear interpolation. Due to the increased data density in time from the zero-padding procedure, the interpolated values are very close to the true values of the data, with discrepancies smaller than the experimental noise level. Linear resampling is then carried out across all pixel values to ensure the image sequence is uniformly distributed in molecular time, setting the stage for subsequent decay time constant and vibrational oscillation analysis.

As mentioned in the main text, separating the exponential decay curve from the oscillatory signal is necessary to obtain an accurate spectrum. Therefore, we first fit the dataset with a multi-exponential curve along the time dimension. This approach is effective because the oscillatory signal is small compared to the exponential decay component. For the results presented in the main text, we fitted the data with a bi-exponential function $y = A\exp\left(-\frac{t}{\tau_1}\right) + B\exp\left(-\frac{t}{\tau_2}\right) + C$, where τ_1 and τ_2 represent the time constants of different exponential decay components. This fitting process is performed either pixel-by-pixel or in a global manner.

In the latter case, which we refer as 'global analysis' in the main text, the three-dimensional dataset $\mathrm{D}(\mathrm{x}, \mathrm{y}, \mathrm{t})$ is reduced to two-dimensions $\mathrm{D}(\mathrm{s}, \mathrm{t})$ by flattening the spatial axis. Subsampling of the spatial dimension can be further applied to reduce the computational time. We use a multi-exponential model to represent the entire dataset:

$$P_{s,k} = \sum_{n=1}^{N} A_{sn} \exp\left(-\frac{t_k}{\tau_n}\right)$$

which can be simplified as $P_{SK} = A_{SN}T_{NK}$, with A an S-by-N matrix that contains 'S' terms per column corresponding to the amplitudes at each spatial point, and T an N-by-K matrix that has 'k' rows representing the temporal response at these spatial points. Since each exponential decay process is independent in our dataset, the T matrix essentially contains N number of exponential term with each $T_{n,k} = \exp\left(-\frac{t_k}{\tau_n}\right)$. This matrix representation separates the multi-exponential model into two parts with one only containing the amplitude information and the other represents the exponential evolution of data in time.

Next, we find the best fit between the experimental data D and the model P by solving A and τ to minimize $\|D - P(A,\tau)\|^2$. We use Moore–Penrose pseudo inverse of T to further simplify this minimization problem with $A_{SN} = D_{SK}T^{+}_{KN}$. Consequently, we only need to solve for τ to minimize $\|D(I - T^{+}T)\|^2$. The amplitude matrix A_{SN} is calculated later with the pseudo inverse of the best fitted T. This process results in 'N' distinct amplitude matrices, each corresponding to a specific exponential decay process; these matrices are then presented as intensity maps for each decay time constant, as described in the main text.

After the exponential decay components are determined, we subtract the fitted results from the original data, $O = D - P$ to isolate the pure oscillatory components, and then apply a Fast Fourier Transform (FFT) along the time dimension to generate hyperspectral maps. We also apply Singular Value Decomposition (SVD) on the spatial-temporal residual $O(s,t)$ to enhance the image quality of the hyperspectral maps. The SVD approach helps isolate the signal from noise by identifying modes that capture spatial patterns and their evolution in time. The dataset O is decomposed to singular vector and singular values following the relation O=USV, where U and V are left and right singular vector matrix, and S is singular values. We retained the first

eight most significant singular values, which contribute predominantly to the signal, and discarded the rest, which are mainly dominated by noise. Figure S4 compares the WSe_2 single-pixel time trace with and without the application of SVD.

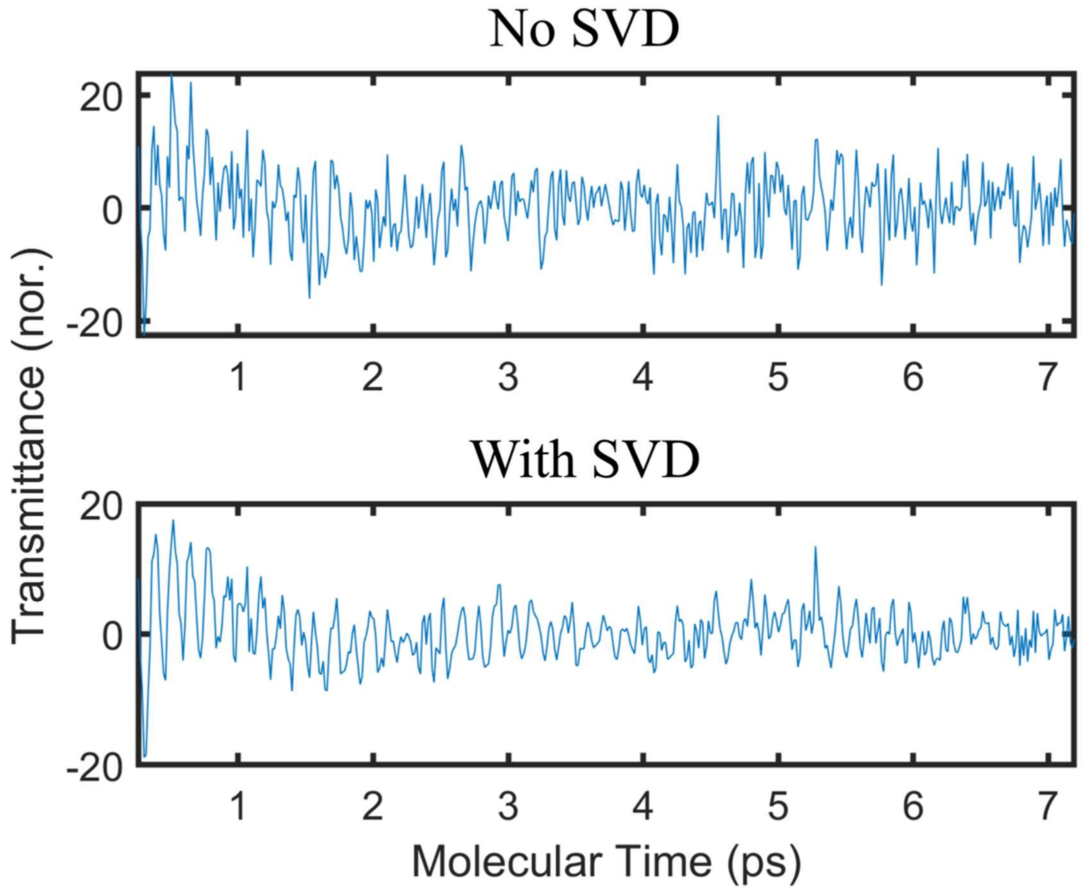

Figure S4: Comparison between the WSe_2 single-pixel time trace without (top) and with SVD (bottom).

We also applied a denoising approach during data analysis (before SVD) to minimize additive noise from the PRISM measurements, utilizing a non-sample region as a reference (Figure S5). Due to the high correlation of noise across the image—an advantage of the wide-field approach—this method was feasible. The approach involved selecting a region with no sample present and multiplying its time trace by a scaling factor to identify the optimal denoising point. The entire image's time trace was then processed by subtracting the scaled reference region's

time trace to effectively reduce noise.

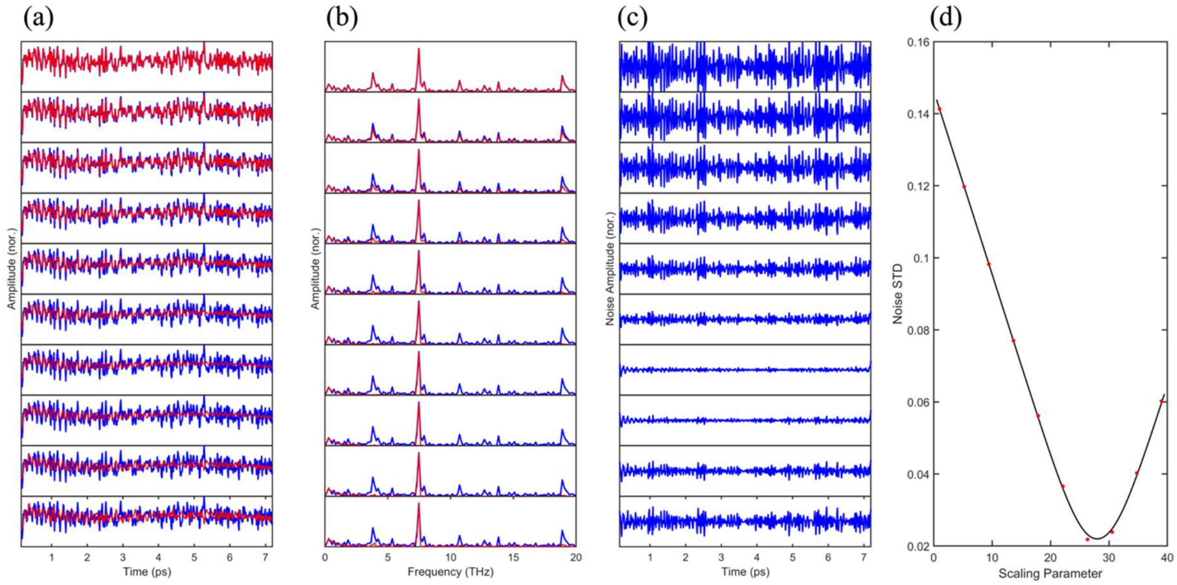

Figure S5: (a) Time traces at single spatial points (blue) compared with denoised curves (red) at different scaling factors. (b) Corresponding spectra before and after denoising. (c) Residual noise signal. (d) Standard deviation of noise, identifying the optimal scaling parameter.

Comparison between PRISM and point-scan spontaneous Raman

Figure S6(a) shows the intensity maps obtained with a point-by-point scan spontaneous Raman setup at the Rayleigh scattering wavelength (0 THz), 0.2 THz, 7.4 THz, and 7.5 THz. As evident, the spatial resolution of the point scan spontaneous Raman measurement is significantly worse than PRISM. Due to the inferior spatial resolution and sensitivity, the few-layer region is less discernable than in the PRISM images. In the low frequency region, the phonon peaks are buried in the Rayleigh scattered and fluorescence background, while for the high frequency region the Raman spectrum detects the E_{2g} and 2LA(M) modes near 7.5 THz[4,5] which broaden the A_{1g} mode. Figure S6(b) presents a comparison of single pixel spectra obtained from both the PRISM (red) and the point-by-point scan Raman (black) within the few-layer and bulk regions. The dashed lines represent the frequencies corresponding to the phonon

intensity maps shown in Figure 2(f). The single-pixel spectrum from the wide-field measurement aligns with the findings from the intensity maps: the few-layer region's interlayer phonon frequency is lower than that of the bulk, and its A_{1g} mode is slighted red-shifted compared to the bulk region. In contrast, the spontaneous Raman spectra exhibit stronger peaks around 4 THz and 11.7 THz, corresponding to the LA(M) and 3LA(M) modes, respectively[6,7]. These non-symmetric modes do not contribute to the DECP signals induced by the coherent pump, which explains why they appear much weaker in the PRISM results[8]. Using PRISM with a pump energy below the bandgap would enable a more balanced intensity between symmetric and non-symmetric modes, as both are excited primarily through ISRS. While non-resonant pump excitations may reduce signal amplitude, compensation can be made by increasing the pump intensity or tuning the probe to the exciton energy for resonance enhancement. Furthermore, the overlaid 7.5 THz intensity maps from both the PRISM and point-scan Raman setups, along with the single line trace comparison in Figure S6(c), clearly demonstrate PRISM's superior spatial resolution. PRISM exhibits a much sharper edge (black line) compared to the point-scan Raman (blue trace), where spatial pixels are more integrated due to the system's limited spatial resolution.

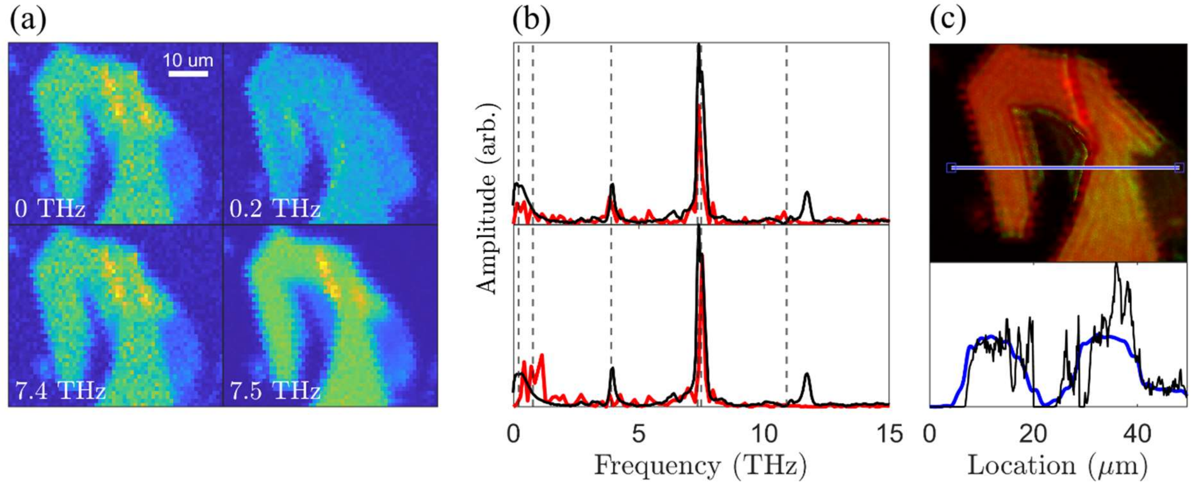

Figure S6: (a) Raman intensity map captured using point-by-point scan spontaneous Raman

setup for comparative analysis. (b) Single pixel spectrum obtained with the wide-field PRISM (red) and point-by-point Raman (black) approach. (c) Overlaid 7.5 THz intensity maps from PRISM (green) and point-scan setup (red), along with the amplitude profile (PRISM: black, point-scan: blue) across the indicated line in the overlaid images.

Comparison between different acquisition speed

Figure S7 compares PRISM results obtained with different acquisition times, ranging from a single scan to 10 averaged scans. The population intensity maps, primarily influenced by exponential decay dynamics, show little change with reduced acquisition time. However, phonon intensity maps are more sensitive to these variations. At the 250 ms acquisition, both population and phonon intensity maps maintain a high SNR, comparable to those in Figure 2 in main text. Reducing the acquisition time further affects the quality of the phonon map, but essential spatial features are still visible down to the 50 ms limit. Faster scanning may be achieved by reducing the scan range at the cost of reduced spectral resolution. These results highlight PRISM's capacity for over one million dynamic traces and spectra per second, with a significant potential for further optimization through hardware and software upgrades, such as faster cameras and delay control schemes, as well as advanced image denoising techniques.

Single-pixel time traces at perovskite microcrystal edge and center

Figure S8 (a)–(b) display the normalized transmittance decay as a function of molecular time for two types of perovskite materials: (a) $MAPbBr_3$ and (b) $MAPb(Br_xI_{1-x})_3$. In both cases, transmittance is measured at two spatial locations: the center (blue line) and the edge (black line) of the crystal. For $MAPbBr_3$, the decay dynamics at the center and edge are quite similar, with only minor variations beyond 2 ps, which are attributed to slower decay components. In contrast, $MAPb(Br_xI_{1-x})_3$ exhibits a more pronounced difference between the edge and center, particularly in the early stages of the decay, where the center shows a faster rise in

transmittance. This indicates that the mixed-halide perovskite exhibits stronger spatial heterogeneity across different regions of the crystal.

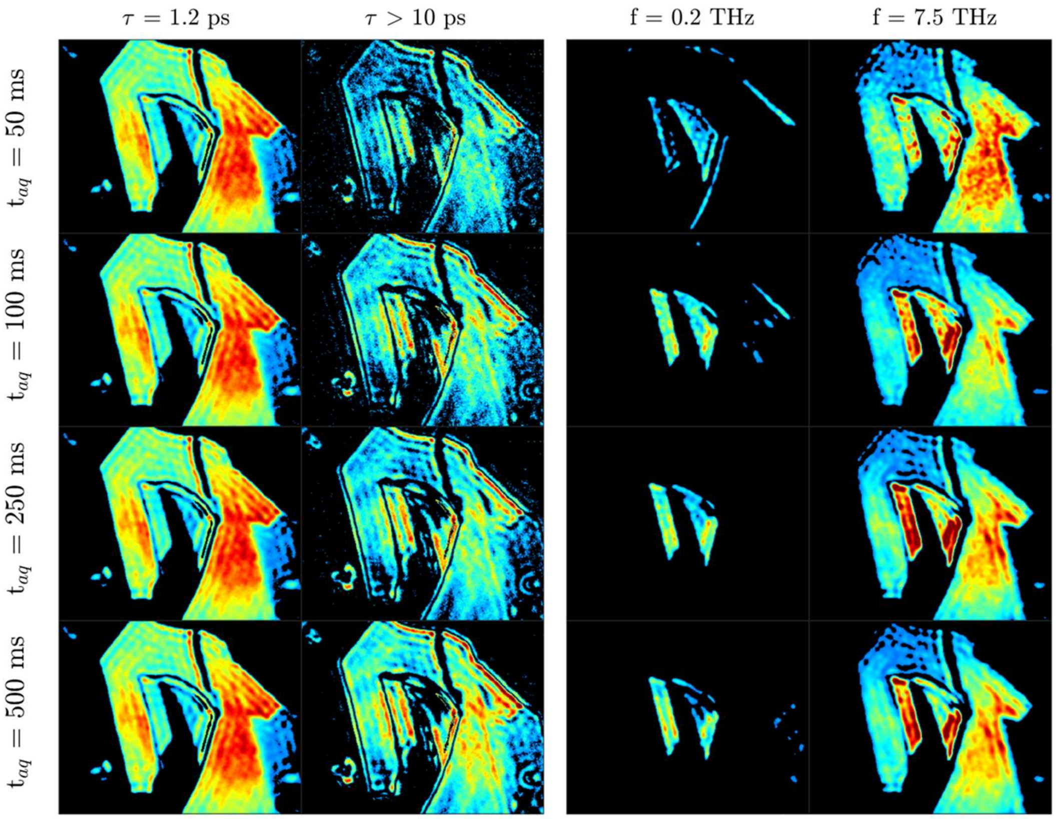

Figure S7: Population intensity maps (first two columns) and phonon intensity maps (last two columns) obtained with varying acquisition time ranging from 50 ms to 500 ms (1 to 10 scans).

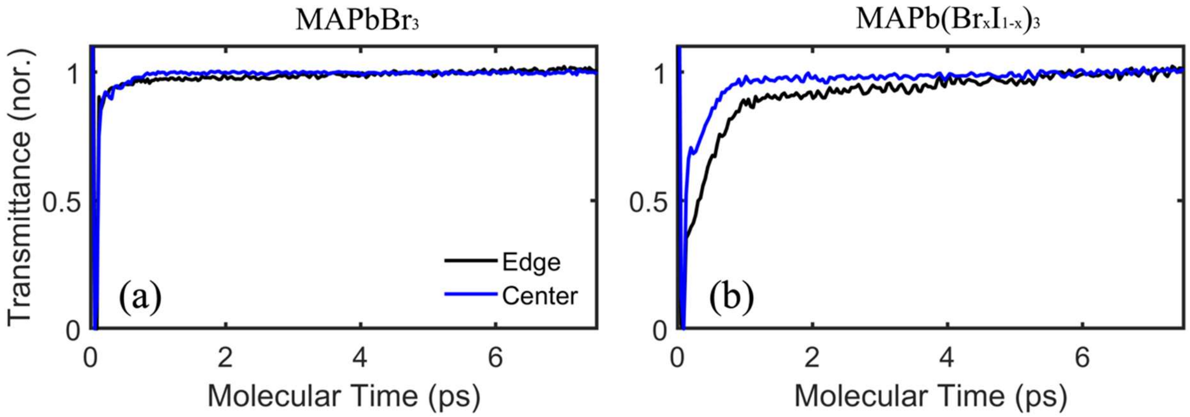

Figure S8: Normalized transmittance decay as a function of molecular time for (a) $MAPbBr_3$ and (b) $MAPb(Br_xI_{1-x})_3$, measured at the center (blue) and edge (black) of the crystal.

Correlation and Principal component analysis

As mentioned in the main text, we performed the correlative analysis of the PRISM results on the WSe_2 flake which illustrates the morphology dependence of electronic decay and vibrational oscillations. Figure S9 reveals the discrepancies between both parameters across different layer numbers. Notably, in the few-layer region, the decay time constant spans a wider range (1.0-1.5 ps) compared to the bulk region (1.2-1.4 ps). This broader range in the few-layer region may result from surface and defect states that introduce additional relaxation pathways. It may also indicate the presence of multiple phonon modes with varying coupling strengths, leading to a broader distribution of decay times

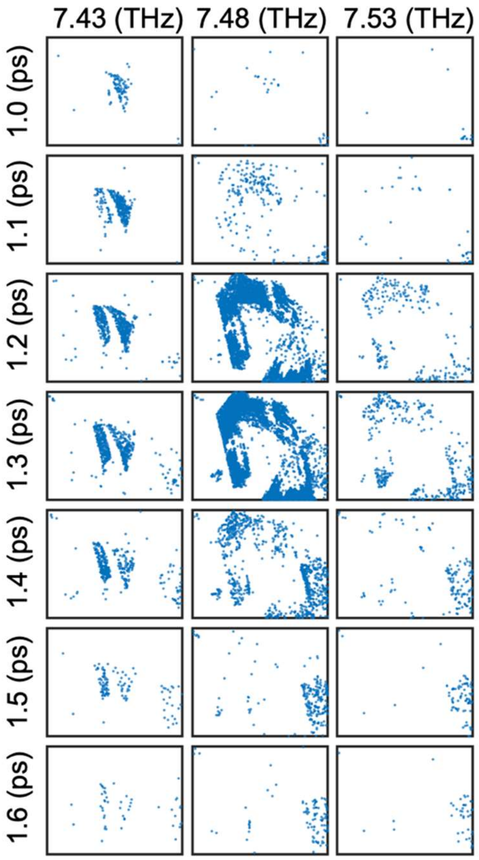

Figure S9: Intensity maps showing different sets of decay time constants and vibrational frequencies.

To perform the PCA analysis, we first flattened the two spatial dimensions of both the PRISM and bright-field data into one-dimensional arrays. We then combined the seven spatially dependent observations into a single data cube, which included two decay components, four frequency peaks (0.2, 0.6, 7.4, and 7.5 THz), and one bright-field transmittance. Since the bright-field image is captured using a CMOS camera, while the time-resolved image sequence is measured with a high-speed camera, an image registration technique is applied to align the two images, ensuring accurate spatial correspondence between the datasets (Figure S10(a)). After structuring the data, PCA was applied to extract the principal components that capture the most significant variations across the sample. These principal components facilitated the clustering of the data, revealing distinct regions within the material. While the 3D plots of these regions are shown in Figure 4(e) of the main text, Figure S10 provides the 2D projection plots of the 3D clusters. The PCA projections along different combinations of components (PC1 vs. PC2, PC1 vs. PC3, and PC2 vs. PC3) illustrate the clear separation of clusters, reflecting variations in vibrational and decay dynamics across the sample.

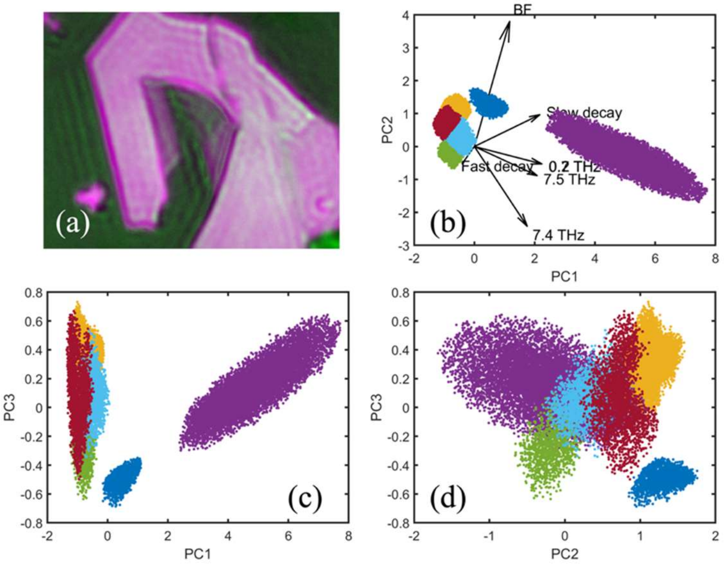

Figure S10: (a) Overlaid bright field (pink) and high-speed camera (green) image. Projection plots of the 3D clusters (main text Figure 4) along PC1 - PC2 (b), PC1 - PC3 (c), and PC2 - PC3 (d).

Effect of Sample Thickness on Signal Level

In the current PRISM setup, both the pump and probe beams reach the sample from the bottom, while the probe light was collected by an objective from the top (main text, Figure 1(a)). This configuration requires the signal to transmit through the sample and reach the detector, thereby imposing a limitation on the measurable sample thickness. Within the heterodyne detection scheme, the integrated pump-probe signal reaching the detector can be approximated as:

$$I_{signal} \sim \chi^3 I_{probe,0} I_{pump,0} \int_0^L e^{-\alpha(z)z} e^{-\beta z}\, e^{-\alpha(z)(L-z)} dz$$

where L is sample thickness, α is the probe absorption coefficient (pump intensity dependent) and β is the pump absorption coefficient. For thin samples $((\alpha + \beta)L \ll 1)$, $I_{signal} \sim I_{probe,0} I_{pump,0} L$, the detected signal increases with increasing sample thickness. For thick samples (samples $((\alpha + \beta)L \gg 1)$, $I_{signal} \sim \frac{I_{probe,0} I_{pump,0}}{\beta} e^{-\alpha_{eff} L}$, with α_{eff} the effective absorption coefficient of probe under pump excitation. In this regime, the signal decays exponentially with increasing sample thickness. The key factors influencing the SNR are the pump and probe beam intensities, the absorption coefficients, and the noise floor of the detector (in this work the high-speed camera). The critical thickness L_c for a detectable signal can be approximated:

$$L_c \sim \frac{1}{\alpha_{eff}} \ln\left(\frac{I_{probe,0} I_{pump,0}}{\beta I_{noise}}\right).$$

For samples thicker than the critical thickness, the signal gets reabsorbed as it propagates, causing the intensity reaching the detector to drop below the noise floor. Figure S11 illustrates the relationship between sample thickness and signal intensity based on the absorption

coefficient[9] of WSe_2. It can be seen that the signal decays to zero at a thickness of approximately 50 μm. For perovskite or other more transmissive samples, the critical thickness increases as the absorption coefficient decreases. This is because lower absorption allows the signal to propagate further through the material before being attenuated.

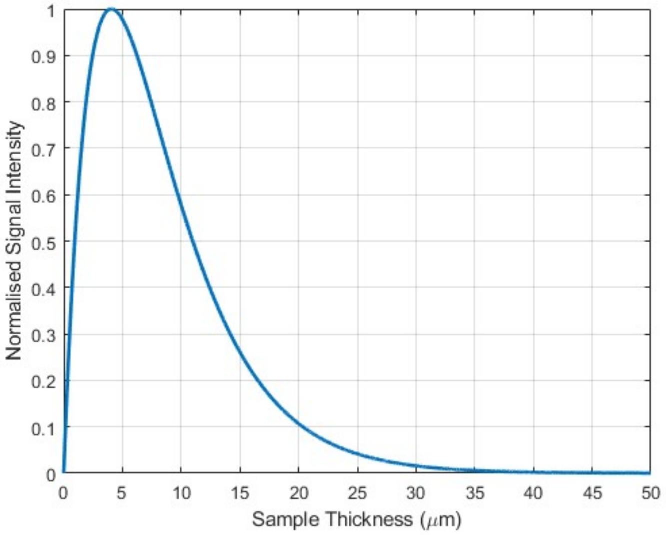

Figure S11: Normalized signal intensity reaching the detector as a function of WSe_2 sample thickness.

References

1. Jeong, T. Y. et al. Coherent Lattice Vibrations in Mono- and Few-Layer WSe2. ACS Nano **10**, 5560–5566 (2016).
2. Vialla, F. & Fatti, N. Del. Time-domain investigations of coherent phonons in van der waals thin films. Nanomaterials **10**, 1–45 (2020).
3. Bresci, A., et al. "Removal of cross-phase modulation artifacts in ultrafast pump–probe dynamics by deep learning." *APL Photonics* 6.7 (2021).
4. Tonndorf, P. *et al.* Photoluminescence emission and Raman response of monolayer MoS2, MoSe2, and WSe2. *Opt. Express* **21**, 4908 (2013).
5. Pan, Y. & Zahn, D. R. T. Raman Fingerprint of Interlayer Coupling in 2D TMDCs. *Nanomaterials* **12**, 1–10 (2022).
6. Del Corro, E. et al. Excited Excitonic States in 1L, 2L, 3L, and Bulk WSe2 Observed by Resonant Raman Spectroscopy. ACS Nano 8, 9629–9635 (2014).
7. Stanford, M. G. et al. Focused helium-ion beam irradiation effects on electrical transport properties of few-layer WSe2: Enabling nanoscale direct write homo-junctions. Sci. Rep. 6, 27276 (2016).
8. Zhang, X. et al. Phonon and Raman scattering of two-dimensional transition metal dichalcogenides from monolayer, multilayer to bulk material. Chem. Soc. Rev. 9, 2757–2785

(2015).
9. Alzaid, Meshal, et al. "Thickness controlling bandgap energy, refractive index and electrical conduction mechanism of 2D Tungsten Diselenide (WSe 2) thin films for photovoltaic applications." Applied Physics A 128, 1-12 (2022).
